# Ophthalmic Telesurgery with a Low-Cost Smartphone Video System for Surgeon Self-Reflection and Remote Synchronous Consultation: A Qualitative and Quantitative Study

**DOI:** 10.1089/tmr.2021.0037

**Published:** 2022-01-31

**Authors:** M. Scott Hickman, William H. Dean, Lila Puri, Sanjay Singh, Rachel Siegel, Daksha Patel

**Affiliations:** ^1^ International Centre for Eye Health, London School of Hygiene and Tropical Medicine, London, United Kingdom.; ^2^ Ad Astra Eye, Lawrence, Kansas, USA.; ^3^ Department of Ophthalmology, University of Cape Town, Cape Town, South Africa.; ^4^ Dhangadhi Netralaya Hospital, Dhangadhi, Nepal.; ^5^ Nepal Eastern Regional Eye Care Programme (EREC-P), Biratnagar, Nepal.; ^6^ Johns Hopkins Bloomberg School of Public Health, Baltimore, Maryland, USA.

**Keywords:** ophthalmology, telesurgery, telemedicine, training

## Abstract

**Summary:**

More than a third of the global burden of blindness is due to cataracts, yet cataract surgery is one of the most cost-effective surgical treatments in medicine. Poor surgical outcomes in many settings remain a major challenge, raising concerns about the quality and efficacy of surgical training. Reflective learning from video recordings of a trainees' surgical performance has a high educational impact and is available routinely for surgical training within high-resource institutions. However, the prohibitive cost and limited portability of current surgical video recording systems make its use problematic in low-resource settings and outreach environments.

**Objective:**

The study's aim was to evaluate the potential of smartphone-captured surgical videos for surgeon learning via self-recording and self-review as well as the potential to support live telesurgical consultation.

**Methodology:**

A quantitative and qualitative methodology was used to explore and describe the utility and acceptance of smartphone videos in two training facilities in Nepal. Twenty surgeries were recorded on the smartphone for surgeon self-review, to assess image quality, and its application to measure performance against the International Council of Ophthalmology (ICO) Ophthalmology Surgical Competency Assessment Rubrics (OSCAR) SICS Rubric. The same system was used to transmit 15 different surgeries live via Skype from Nepal to an ophthalmologist surgical trainer in South Africa to evaluate the feasibility of live consultation.

**Findings:**

Overall video quality was described as high in 65% and moderate in 35% for the videos recorded for self-review. In the surgeries streamed via Skype, quality was described as high in 92.9% and moderate in 7.1%. There were no instances where the video quality was described as poor. The video quality was good enough that the surgeons could measure against ICO-OSCAR rubric in all cases.

**Discussion:**

The video quality of smartphone-captured surgical videos was found to be high and gained acceptance for reflective teaching and learning purposes. The extended telesurgical potential and portability of the smartphone enables use across many settings. More studies over a longer period are needed to determine how they can support training and learning in cataract surgery.

## Background

Cataract surgery is one of the most successful surgeries in the world.^1^ There are 36 million people blind in the world with more than one-third of the blindness due to cataracts,^2^ and most of those affected are in low and middle income countries (LMIC). Increasing cataract surgeries across health systems in low-resource settings is linked closely with the availability of trained human resources in eye care, particularly in rural areas. In Africa, it is estimated that there are 2.9 eye care professionals for a 1 million population.^3^ This shortage relays the urgent need to increase training and strengthen services to address the growing backlog of cataract surgical cases^4^ due to increasing longevity and population growth.^5^

Surgical quality (measured as visual outcome) is as important as quantity of surgeries. World Health Organization (WHO) guidelines for eye care programs state that poor outcomes (postoperative visual acuity <6/60 with best correction) should aim to be <5%. However, reports of poor surgical outcomes after cataract surgery have ranged from 22% in Kenya,^6^ 25% in China,^7^ 30% in Zambia,^8^ and up to 38% in Libya.^9^ Poor outcomes can be traced to surgical techniques, patient-related factors, or comorbidities not previously identified due to the advanced nature of cataracts often seen, or due to postoperative refractive errors. A review from Libya shows that after discounting all other causes of poor outcome, more than 24% were linked directly to surgical technique.^9^

It is common for cataract surgery training to use a mentor and apprentice teaching method.^10^ Microsurgical skills transfer is demanding for teaching and learning, as the development of fine manual dexterity is guided through performance observations. In practice, application of this training model has been recognized to have multiple challenges and has evolved to include a wide range of training tools in high-resource settings.^11^ Growing sophistication of tools including videos, simulators, wet labs, and tele-mentoring tools for skills development and patient safety build the confidence and competency of the junior surgeon in an environment safe for the patient.^10^

In the 1960s, Fitts and Posner described a model for the acquisition of motor skills learning in three stages: cognitive, associative, and autonomous.^12^ In the cognitive stage, the learner gains supportive knowledge and ability to perform a skill through observation and mentoring. In the associative stage, solutions are explored by evaluation and comparison of patterns and processes within their own technique and to manage complications. In the autonomous stage, the student develops an in-depth, almost intuitive, perspective of the process and measures that can be applied, requiring little cognitive effort and can perform motor tasks automatically.

A study on manual small incision cataract surgery (MSICS) training in low-resource settings revealed that most complications occur during the trainees' first month of surgery and they remained highly dependent on a supervisor's assistance.^13^ The International Council of Ophthalmology (ICO) has developed Ophthalmology Surgical Competency Assessment Rubrics (OSCAR) for ophthalmic surgical procedures including SICS to grade a surgeon on their competency on the different steps of MSICS and to support a teaching and learning progression.

Video technology has the potential to enhance cataract surgical training for the observer and the observed. Video capture of surgical procedures in ophthalmology began in the 1970s, as described next by Dr. Robert Osher in 2011:

“Ophthalmic surgery cannot be learned from the podium. Nor can it be mastered from a textbook. Although there is nothing as valuable as sitting at the microscope and actually performing a procedure, it is not so easy on a trainee's coronaries, and inexperience is not always fair to the patient. How, then, can residents and fellows learn to operate? The obvious answer is by watching videos.”^14^

The opportunity for a reflective approach to the practice raises the quality of the learning experience. In many LMIC training settings, the implementation of this technology is prohibitive as the cost of video capture technology is a limiting factor and average U.S. systems cost more than US$20,000.

The growing availability of high-quality smartphone cameras has the potential to become a low-cost educational tool for low-resourced training facilities. The same is true for the transfer of this material via the Internet for review by a surgical trainer (tele-mentoring) or for peer consultation on complex cases. High-cost methods of video teleophthalmology exist, but a low-cost service such as Skype (Skype Communications, Luxembourg) increases accessibility. Portability of this technology further supports the potential for use in outreach and rural settings where surgical services are provided closer to the patients.

Although there have been many studies looking at the reliability of teleophthalmology, especially diabetic screening and slit lamp images,^15–20^ to our knowledge there have been few on synchronous surgical teleophthalmology. Ye et al. have shown that it is feasible to transmit good quality surgical and slit lamp images using an Apple iPhone (Apple, Cupertino, California) from China to Florida using a broadband Internet connection.^21^ Camara et al. used real-time telementoring to successfully remove a lateral orbital tumor with an oculoplastic surgeon in a remote location guiding the operating general ophthalmologist.^22^

The objective of this study was to assess the technological feasibility and acceptance of using an iPhone 7 Plus (Apple, Cupertino, California) and an Orion SteadyPix Pro Smartphone/Camera Mount (Orion, Watsonville, California) to review recorded surgical performance against the ICO OSCAR SICS scale by cataract surgeons. In addition, it tested the function of the system across broadband Internet video transmission via Skype to review surgical performance remotely against the ICO OSCAR SICS scale.

An ophthalmic surgeon, especially a recent graduate, will be confronted with unexpected situations where they are unsure of the best course of action. Accessing a more experienced doctor in real time, who can adequately see the surgical situation remotely, and communicate possible interventions through a safe, easy, and affordable manner has the potential to improve patient safety and outcomes while the patients are still in the operating theatre. Further, a training eye surgeon performing supervised cataract surgery will be able to record their surgical performance affordably on a smartphone. Critically, on subsequent review of the video they can engage in “reflective learning” and self-appraisal, powerful educational experiences.

## Materials/Patients and Methods

The study design is descriptive and used both qualitative and quantitative methods of data collection. The study data collection period was 2 weeks in June of 2017. Ethical approval was obtained from the ethics committees of the London School of Hygiene and Tropical Medicine, Eastern Regional Eye Care Programme (EREC-P) at Sagarmatha Choudhary Eye Hospital (SCEH) in Lahan, Nepal, and Biratnagar Eye Hospitals (BEH) in Biratnagar, Nepal (LSHTM Ethics reference number: 13846-1). Participants were informed about the details and nature of the study and invited to consent to participation.

The study location was an established high-volume, high-quality training center, EREC-P in Eastern Nepal. Synchronous teleophthalmic surgical videos sharing was with the University of Cape Town, South Africa.

**FIG. 1.**
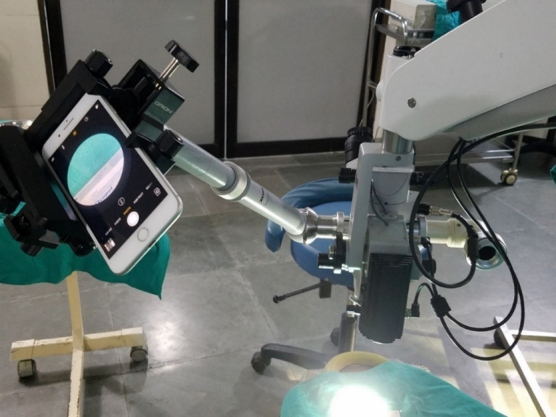
Setup of smartphone recording system/Skype teleophthalmology system.

An example of the recording setup is seen in Figure 1.

The recording system components were all commercial products and assembled on the operating microscope with the beam splitter and connected to the Internet along with Skype (Table 1). The smartphone used was an iPhone 7 Plus running iOS 10 with 256 GB with a 12-MP camera by using 30 frames per second and uncompressed videos. The cost was ∼1000 USD and was purchased with funds from the Hooper Scholarship through the International Centre for Eye Health and the London School of Hygiene and Tropical Medicine Bench Fees Fund (Table 2).

**Table 1.** Equipment for Smartphone Video Recording System and Specific Instruments Used in This Study

General equipment	Specific equipment used for this study
Smartphone with ability to take pictures at 5 MP minimum	iPhone 7 Plus running iOS 10 with 256 GB with 12-MP camera
Smartphone adapter that can fit onto a surgical microscope eyepiece	Orion SteadyPix Pro Smartphone/Camera Mount
Surgical microscope with the ability to fit a beam splitter and an observing microscope	Zeiss S7 ophthalmic microscope
Beam splitter	Carl Zeiss beam splitter
Observing microscope	12.5 × Carl Zeiss Eye Piece Ocular 305543–9901 attached to a Carl Zeiss slit lamp teaching scope
Viewing station (can be a smartphone itself)	For Self-review: HP Pro × 2 laptop For Skype review: MacBook Air
WiFi router	

**Table 2.** Cost of Smartphone Video Recording/Teleophthalmology System Used in Study Compared with Cost of High-Income Country Video Recording System

Equipment in study	Cost in USD
Smartphone: iPhone 7 Plus	992.25
Orion SteadyPix Pro adapter	59.99
	Total: 1052.24 USD
Equipment in high-income setting	
Beam splitter	3681.61
Video lens	2915.32
Stereo observation tube	7159.54
Inclined Binocular Tube	4824.07
2 Widefield eyepieces	2046.18
2 Sleeves for eyepieces	74.88
	Total: 20701.60 USD

### Methods used in the study

Twenty-one out of 32 (65.6%) of total surgeons at EREC-P consented to participate in an online survey about their cataract surgery training experiences before using the smartphone video option. This included 12 trainees, which included 10 fellows and 2 residents, and 9 attendings. For the intervention review, 20 patients consented to have their operations anonymously recorded. Ten surgeries were recorded each at Lahan and Biratnagar. Of these 20 surgeries, there were 4 teaching attendings and 3 fellows in training who reviewed the video quality of their smartphone captured surgery with the principal investigator.

The smartphone recorded cataract surgery videos, and the assessments of their video quality were compared with the ICO OSCAR SICS framework by the operating surgeon. In addition, 15 different patients and 2 attending surgeons, 1 each at Lahan and Biratnagar, consented to have their operation recorded and sent for review by 1 ophthalmologist remotely in South Africa grading the video in real time via Skype, a U.K. based ophthalmologist in South Africa familiar with the OSCAR scale.

Each surgeon marked the quality of the video captured as high, moderate, or low on their ability to grade their surgical steps using the ICO SICS OSCAR rubric. *High quality* was defined as being able to review all the steps of the surgery, *moderate* was being able to review almost all the steps but not all, and *poor* was not able to see their surgery. The key steps reviewed were: overall video quality, sclera-corneal tunnel, corneal entry, capsulorrhexis, hydrodissection, nucleus prolapse, nucleus extraction, cortical removal, intra-ocular lens (IOL) insertion, and wound closure.

Postsurgical interviews with the three trainee fellow surgeons and four attending trainer surgeons were done by the principal investigator in a semi-structured format at the end of the day. The interviews were recorded and transcribed to determine the likes, dislikes, challenges encountered during the image review and their acceptability of the system to aid surgical training in the local setting. Themes were attempted to be seen with immersion of the material.

Finally, extraction of any possible meaning of the smartphone recording video experience was attempted. Qualitative methodology was selected, as it provided flexibility to ask questions beyond the obvious completion status of the task, and gain and in-depth understanding of the underlying perception about the technology and its potential in the training process.

## Results

Overall video quality was graded as high in 70% and moderate in 30% of responses from the operating surgeons. Nucleus extraction, IOL insertion, and wound closure were rated high by 100% of respondents. The scleral tunnel video quality was rated high by the trainers in 60% of the cases, and moderate in 40%. Hydrodissection was rated as 50% high and 50% moderate. The capsulorrhexis quality received the lowest video rating with 45% rating high, and 55% moderate. When asked whether they could potentially grade all steps of their surgery from novice to competent via the ICO OSCAR SICS rubric, the surgeons reported they could grade it 100% of the time. None of the surgical steps were rated as poor video quality (Table 3).

**Table 3.** Combined Self-Review Data of the Fellows at Biratnagar and Consultants at Lahan

Biratnagar fellow and Lahan consultant video results	High video quality, ***N*** (%)	Moderate video quality, ***N*** (%)	Poor video quality, ***N*** (%)	Yes, could rate surgical step, ***N*** (%) in ICO rubric	No, could not grade surgical steps, ***N*** (%)
Surgical steps					
Overall video quality	13 (65.0)	7 (35.0)	0	n/a	n/a
Sclerocorneal tunnel	12 (60.0)	8 (40.0)	0	20 (100.0)	n/a
Cornea entry	17 (85.0)	3 (15.0)	0	20 (100.0)	n/a
Capsulorrhexis	9 (45.0)	11 (55.0)	0	20 (100.0)	n/a
Hydrodissection	10 (50.0)	10 (50.0)	0	19 (100.0)	n/a
Prolapse of nucleus	17 (85.0)	3 (15.0)	0	20 (100.0)	n/a
Nucleus extraction	20 (100.0)	0	0	20 (100.0)	n/a
Cortical removal	17 (85.0)	3 (15.0)	0	20 (100.0)	n/a
IOL insertion	19 (95.0)	1 (5.0)	0	20 (100.0)	n/a
Wound closure	18 (90.0)	2 (10.0)	0	20 (100.0)	n/a

ICO, International Council of Ophthalmology; IOL, intra-ocular lens.

All trainee surgeons felt this technology was suitable for their learning process and recommend its use. All felt it was either very helpful (90%) or helpful (10%) to use the recording system to improve their surgical education and performance.

The process of telementoring from the reviewer in South Africa from Nepal was described as high video quality in 92.9% of the cases and as moderate in 7.1%. When breaking down the steps of the surgery, those that scored less than high in 80% of the surgeries included the capsulorrhexis (high 53.3% and moderate 46.7%), hydrodissection (high 50%, moderate 50%) and irrigation and aspiration technique (high 73.3% and moderate 26.7%). These results confirm the high quality of the images and the potential of extending this system to remote settings (Table 4).

**Table 4.** Skype Video Results of Images Sent from Nepal to South Africa to a U.K.-Based Ophthalmologist

Skype video results of SCEH and BEH	High video quality, ***N*** (%)	Moderate video quality, ***N*** (%)	Poor video quality, ***N*** (%)	Yes, could rate surgical step, ***N*** (%)	No, could not grade surgical steps, ***N*** (%)
Surgical steps					
Overall video quality	13 (92.9)	1 (7.1)	0	n/a	n/a
Sclerocorneal tunnel	14 (93.3)	1 (6.7)	0	15 (100.0)	n/a
Cornea entry	14 (93.3)	1 (6.7)	0	14 (100.0)	
Capsulorrhexis	8 (53.3)	7 (46.7)	0	13 (86.7)	1 (6.7)
Hydrodissection	7 (50.0)	7 (50.0)	0	14 (100.0)	n/a
Prolapse of nucleus	15 (100.0)	0	0	14 (100.0)	n/a
Nucleus extraction	15 (100.0)	0	0	15 (100.0)	n/a
Cortical removal	11 (73.3)	4 (26.7)	0	15 (100.0)	n/a
IOL insertion	15 (100.0)	0	0	15 (100.0)	n/a
Wound closure	14 (100.0)	0	0	13 (100.0)	n/a

BEH, Biratnagar Eye Hospitals; SCEH, Sagarmatha Choudhary Eye Hospital.

### Qualitative analysis of smartphone video technology experience

Interviews with the participants were transcribed and after immersion were analyzed for themes. Two key themes emerged, highlighting the challenges and potential of the system:

- Practical concerns: Image magnification and technical difficulties
- Potential of the system

The smartphone image had a smaller field of view compared with the operating microscope image, creating problems with centering the image size. Setting up and maintaining the functionality of the system caused technical delays and frustrations from all of the interviewees.

The Biratnagar 1 fellow stated:

*What I liked is that you could see your video. What I didn't like was that there were a lot more processes to setting up, and the exposure and instrument setting. And you had to take care of the brightness, and contrast, and centration of the patient…It would be better if it was automatic.*

All the interviews revealed the possibility of using the system to teach others locally at EREC-P, for personal learning and self-review, as well as disseminating local surgical expertise to a wider global audience. A consultant from Lahan noted:

*By directly showing them (the residents) the procedure and not just by using bookish language…we can teach the residents and fellows by showing them each step. I think seeing is better than listening.*

In addition, some surgeons in this high-volume setting in Nepal had performed ∼30,000 cataract surgeries and had never seen themselves operate. Reflecting on each stage of their skillset afforded a new appraisal of and pride in their performance described by the Biratnagar 2 fellow:

*It was really amazing; I have never seen my own recording before. I have seen other videos but seeing my own video for the first time was really amazing. I thought I would not be doing it the correct way on the video recording, but it was fine, it was more than I expected.*

The Biratnagar fellow 3 used these words on seeing themselves operate for the first time: *“I am overwhelmed and happy.”*

From an institutional perspective, sharing training and local surgical techniques across their partners was regarded as a new opportunity for EREC-P. Examples include spreading their use of the fishhook technique and linear capsulotomy, or the management of complex surgeries.

For example, the Biratnagar fellow 2 said:

*Yes, you can use it for teaching cataract surgery… and Skype and share videos. You can share with your colleagues in a different hospital. We are using the fishhook technique and they can learn using these videos on how to use a fishhook. It is a good way to share our skills.*

The Biratnagar fellow 2 also noted:

*I think this will be very beneficial and adoptable with the smartphone, so that you can learn… (and) use your experience for a larger audience with this technology.*

## Discussion

Strengthening training for cataract surgery is a cornerstone in eliminating avoidable cataract blindness. As such, various regulating bodies have introduced new surgical competencies. The International Curricula of Ophthalmic Education from the Task Force ICO developed 15 topics on resident education in an effort to move from an apprenticeship model to a curriculum-based one.^23^ The Accreditation Council on Graduate Medical Education has six core competency outcomes of resident education.^24^ There has also been a seventh competency adopted in surgery, including by the American Board of Ophthalmology.^25^

New frameworks have been developed to measure resident surgical competency in response to this shift.^26^ An Objective Assessment of Skills in Intraocular Surgery (OASIS) was developed to objectively assess surgical competency with factual data based on phacoemulsification.^25^ This evaluation collected information, including preoperative workup and planning, surgical competencies, and postoperative care. Another model described an ophthalmic wet laboratory to teach and assess resident surgical competence,^27^ the latter being involved in a reduction in complication rates.^28^ The ICO has also published an OSCAR for SICS.^29,30^

The challenges faced in low-resource settings include low trainer to trainee ratios, managing high volumes of patients, and the need to train the residents to manage across a wide range of settings, including outreach in rural centers. The constant need to review the knowledge, skills, and reflective application necessary for reducing complications, especially in the cognitive Fitts Posner stage, is central to the steep learning curve in microsurgical skills development.

Video during this early period allows for reflective learning and supports peer discussion to enhance individual surgical skills, particularly during the cognitive stage. It has been shown that complications such as vitreous loss during phacoemulsification reduce at about 80 cases, from 5.1% before 80 cases to 1.9% afterward.^31^ A similar study with MSICS showed that most complications occur during the first month of surgery as well as a supervisor's assistance being most common during the first month of surgery.^32^

A smartphone-based tele-surgery system is an economical alternative to the standard surgical video system that has been used for decades in high-resource settings to record and review surgeries for improvement. Technical difficulties are present, yet they are surmountable as the technology continues to develop. High-quality images and the potential for self-review with a supportive trainer offer a new perspective on improving training from outside the stressful operating theater, which is crucial when wet labs or virtual reality simulation surgery are unavailable. Cost comparison of the smartphone systems used in this study (1052.24 USD) with a surgical video recording system of 20701.60 USD suggests that this is a feasible option in resource-limited settings.

As identified in this study, even in high-volume training centers, there was a recognition of the value of self-reflection and personal satisfaction in one's surgical performance that this system can address. Systematic visualization of distinct steps, through a rubric or a standardized assessment format, can strengthen the development of a feedback mechanism for the mentor and peer reflection after the procedure and not only during the procedure, as is the case in the present apprentice-based training format. Video playback and reflection supports the mastery of surgical details, especially in the absence of simulation training.

As technology continues to develop, the process of recording and sending videos in real time could have a significant impact on global ophthalmic education. The system described would allow surgeons who have left their training program recently and are placed by themselves in remote areas to receive mentorship and surgical advice in real time. In the future, this equipment could be considered standard practice for setting up a recent graduate in a remote location. In addition, more experienced surgeons could receive help with a new procedure or complicated surgery. Nonphysician cataract surgeons, found in many parts of the world including Kenya, could also receive mentoring in real time.

Due to the lack of human resources overall, many LMIC training programs have a shortage of available consultants to teach residents or an absence of sub-specialists for referral of complex cases. One potential solution to this dearth of human resources is the collaboration with other residency programs, such as those found in the United Kingdom or United States with the Vision2020 LINKS Programme.

These cross-institution programs involving teleophthalmology with surgical videos, clinic referrals, teaching via slit-lamp images or diagnostic images, and lectures have the potential to expand both training institutions' educational experiences. Medical and legal issues such as patient confidentiality in using such a system should be considered for each institution-to-institution relationship.

## Conclusions

This study illustrates the initial acceptability and utility of a simple, affordable, and reliable video recording system for surgical procedures in the ophthalmic operating theater. Trainee eye surgeons can record their surgical performance by using a smartphone or send them to another surgeon in real time for review. This system will enable reflective learning when reviewing a procedure. It can be a powerful educational experience when combined with self-assessment using the ICO SICS OSCAR Rubric and feedback from a surgical trainer.

Although we have attempted to describe the surgical video quality and the difficulties in seeing parts of surgery mentioned earlier, it is best to view the videos for oneself. Video examples have been placed on YouTube and are accessible via the links next.

https://youtu.be/9OCct7Cl19g

https://youtu.be/t677eOKuiyI

One limitation of the study is that it does not measure whether it improves patient outcomes. It is also limited to one ophthalmic training program with limited number of doctors and patients involved, as well as using one ophthalmologist as a Skype reviewer as a potential source of bias. The cost of the smartphone and the adopter device may also be a limiting factor, especially if a beam splitter and observing scope needs to be purchased as well.

The setup of the recording device is somewhat difficult, and it takes time and effort to learn and maintain. The portion of the study involving Skype is dependent on the speed of the Internet connection, which will vary throughout LMIC in its speed and availability.

## Abbreviations Used

BEH: Biratnagar Eye Hospitals
EREC-P: Eastern Regional Eye Care Programme
ICO: International Council of Ophthalmology
IOL: intra-ocular lens
LMIC: low and middle income countries
MSICS: manual small incision cataract surgery
OASIS: Objective Assessment of Skills in Intraocular Surgery
OSCAR: Ophthalmology Surgical Competency Assessment Rubrics
SCEH: Sagarmatha Choudhary Eye Hospital
VA: visual acuity
WHO: World Health Organization
